# Case Report: EBV Chronic Infection and Lymphoproliferation in Four APDS Patients: The Challenge of Proper Characterization, Therapy, and Follow-Up

**DOI:** 10.3389/fped.2021.703853

**Published:** 2021-08-27

**Authors:** Beatrice Rivalta, Donato Amodio, Cinzia Milito, Maria Chiriaco, Silvia Di Cesare, Carmela Giancotta, Francesca Conti, Veronica Santilli, Lucia Pacillo, Cristina Cifaldi, Maria Giovanna Desimio, Margherita Doria, Isabella Quinti, Rita De Vito, Gigliola Di Matteo, Andrea Finocchi, Paolo Palma, Antonino Trizzino, Alberto Tommasini, Caterina Cancrini

**Affiliations:** ^1^Research Unit of Primary Immunodeficiencies, Immune and Infectious Diseases Division, Academic Department of Pediatrics (DPUO), Bambino Gesù Children's Hospital, IRCCS, Rome, Italy; ^2^Chair of Pediatrics, Department of Systems Medicine, University of Rome “Tor Vergata”, Rome, Italy; ^3^Research Unit of Clinical Immunology and Vaccinology, Academic Department of Pediatrics (DPUO), Bambino Gesù Children's Hospital, IRCCS, Rome, Italy; ^4^Department of Molecular Medicine, Sapienza University of Rome, Rome, Italy; ^5^Pediatric Unit, IRCCS Azienda Ospedaliero-Universitaria di Bologna, University of Bologna, Bologna, Italy; ^6^Pathology Unit, Department of Laboratories, Bambino Gesù Children's Hospital, Rome, Italy; ^7^Department of Pediatric Hematology and Oncology, ARNAS Civico Di Cristina and Benfratelli Hospital, Palermo, Italy; ^8^Institute for Maternal and Child Health, IRCCS Burlo Garofolo, Trieste, Italy; ^9^Department of Medicine, Surgery and Health Sciences, University of Trieste, Trieste, Italy

**Keywords:** APDS, PI3Kdelta kinase, EBV, lymphoproliferation, p110δ, p85α, PI3K-AKT-mTOR

## Abstract

Activated PI3K-kinase Delta Syndrome (APDS) is an autosomal-dominant primary immunodeficiency (PID) caused by the constitutive activation of the PI3Kδ kinase. The consequent hyperactivation of the PI3K-Akt-mTOR pathway leads to an impaired T- and B-cells differentiation and function, causing progressive lymphopenia, hypogammaglobulinemia and hyper IgM. Patients with APDS show recurrent sinopulmonary and chronic herpes virus infections, immune dysregulation manifestations, including cytopenia, arthritis, inflammatory enteropathy, and a predisposition to persistent non-neoplastic splenomegaly/lymphoproliferation and lymphoma. The recurrence of the lymphoproliferative disorder and the difficulties in the proper definition of malignancy on histological examination represents the main challenge in the clinical management of APDS patients, since a prompt and correct diagnosis is needed to avoid major complications. Targeted therapies with PI3Kδ-Akt-mTOR pathway pharmacologic inhibitors (i.e., Rapamycin, Theophylline, PI3K inhibitors) represent a good therapeutic strategy. They can also be used as bridge therapies when HSCT is required in order to control refractory symptoms. Indeed, treated patients showed a good tolerance, improved immunologic phenotype and reduced incidence/severity of immune dysregulation manifestations. Here, we describe our experience in the management of four patients, one male affected with APDS1 (P1) and the other three, a male and two females, with APDS2 (P2, P3, P4) presenting with chronic EBV replication, recurrent episodes of immune dysregulation manifestations and lymphomas. These cases highlighted the importance of a tailored and close follow-up, including serial endoscopic and lymph nodes biopsies control to detect a prompt and correct diagnosis and offer the best therapeutic strategy.

## Introduction and Review of Literature

Activated PI3K-kinase Delta Syndrome (APDS) is an autosomal dominant primary immunodeficiency (PID) resulting from a gain of function (GOF) mutation in PIK3CD gene (1, 2) encoding the leukocyte-restricted catalytic subunit p110δ or from a splice-site mutation in the PIK3R1 gene (3, 4) encoding the ubiquitously expressed regulatory subunit (p85α, p55α and p50α) of the phosphatidylinositol 3-kinase delta (PI3Kδ) complex. The hyperactivation of the PI3Kδ leads to the constitutive activation of the Akt-mTOR pathway, measurable *in vitro* as AKT and ribosomal S6 kinase hyperphosphorylation in B and T lymphocytes. The PI3K-AKT-mTOR signaling pathway is involved in the metabolism, differentiation, proliferation, growth, survival and motility of immune cells (5, 6). Its hyperactivity leads to progressive lymphopenia with a defective T- and B-cells differentiation and function (7–12). Defects of NK cells subsets, in terms of number, maturation and cytotoxic function, and of the myeloid compartment were also reported in some APDS patients (7, 13, 14).

In this condition, T cell phenotype is characterized by a reduction of CD4+ and CD8+ naïve T cells and an expansion of terminally differentiated CD8+CCR7-CD45RA+ effector memory T cells (1, 2, 4, 8, 11, 15–17). The frequency of regulatory cells (T-reg) is reported in the normal range, although an impaired ability to express FoxP3 upon stimulation *in vitro* has been reported (18). Therefore, further studies are necessary to determine the role of PI3K-AKT-mTOR pathway in T-reg development and function and their activity in APDS (19, 20). Peripheral T follicular helper cells (pTfh) are usually increased and they show a Th1 phenotype with increased PD1, CXCR3 expression and IFNγ secretion, suggesting a compromised Tfh-mediated B cell help (germinal center reaction) (21, 22). This altered Tfh differentiation seems to be ascribable to different mechanisms not only related to the hyperactivation of the PI3K-AKT-mTOR pathway (18). B cells are usually reduced, particularly memory (CD19+CD27+) and switched memory B cell subset (CD19+CD27+IgD-IgM-), with an expansion of transitional (CD19+CD38 hi IgM hi) and CD21low B cell (23). Immunoglobulin production and vaccine response are usually impaired with hypogammaglobulinemia and increased or normal IgM (9, 15, 16, 24, 25).

Patients develop recurrent sinopulmonary and respiratory tract infections, chronic herpes viruses infections and in some cases persistent granulomatous skin lesions associated with BCG vaccination (26). Immune dysregulation manifestations are among the most common features, especially cytopenia, arthritis and inflammatory enteropathy. Persistent splenomegaly and lymphoproliferation is a hallmark of the disease, potentially involving airways and the gastrointestinal tract, with increased susceptibility to malignant degeneration (15, 16, 27–29).

Failure to control Herpes viruses such as EBV, characterize both APDS and other PIDs with T and NK cells impairment as combined immunodeficiency (CID). In these conditions, EBV infection may lead to different complications as lymphoproliferation, malignancy (30), trigger immune-dysregulation (31) or hemophagocytic lymphohistiocytosis (HLH) (32–34). In other PIDs, EBV infection is associated with an increased risk for B-cell lymphoproliferative disorders and lymphomas due to an impairment in T-cell activation (as RASGRP1, MAGT1 and ITK), DNA metabolism essential for the expansion of activated antigen-specific T cells (CTPS1) or co-stimulatory pathways (CD70, CD27, and TNFRSF9) necessary for elimination of proliferating EBV-infected B cells. Others mechanisms include a defective cytotoxic killing of EBV-infected B cells leading to a protracted T-cell expansion and activation resulting in HLH. Among these are described mutations in the SH2 domain protein-1A (SH2D1A) gene encoding SAP (SLAM-associated protein) responsible for XLP1 or mutations in XIAP (Inhibitor of apoptosis, X-linked) gene (XLP2) (10, 32, 35).

In APDS patients, the persistence of EBV replication can be mainly explained by the impaired cytotoxicity of NK and CD8+ T cells. Despite a normal or high frequency of EBV-specific CD8+ T cells, this subset shows an immunosenescent/exhausted phenotype, with clonal expansion inability, defective killing of EBV-infected targets and increased susceptibility to restimulation-induced cell death (RICD) or engagement of CD95. *In vitro* NK cytotoxicity assay against target cells is impaired due to reduced conjugate formation with infected target cells and inadequate cell machinery activation required for killing. Furthermore, the expansion of transitional B cells which may represent one of the EBV reservoirs and the impaired antibody responses, may contribute to viral persistence (7, 10, 13, 36).

The impaired EBV clearance and the intrinsic B cell proliferation contribute to lymphoma pathogenesis. Persistent B cell proliferation is maintained by the constitutive activation of PI3Kδ signaling, partly activated by the expression of viral EBV proteins, as the latent membrane protein 1 (LMP1) (10, 37). Moreover an impaired NK activity could also be responsible for a reduction of tumor surveillance (7). One-fifth of APDS patients with a chronic EBV develop lymphoma during follow-up, but the majority of lymphoma reported are not EBV positive highlighting the intrinsic susceptibility of APDS1/2 to malignant degeneration (15, 16, 27, 38). Few cases of HLH are reported in APDS patients, presumably explained by the profound impairment of T cell function (39–41). Probably, the tendency of APDS T-cells to apoptosis may protect against HLH development in these patients (12).

The architecture of proliferating lymph nodes is usually preserved and characterized by prominent hyperplastic germinal centers with absent or markedly reduced mantle zone. Hyperplasia of follicular CD4+PD1+ cells and PD1+CD57+ senescent T cells accumulate in both T and extrafollicular areas. Aggregates of monocytoid B cells, a reduction of IgG+ plasma cells and expansion of IgM plasma cells are also described (2, 15). These anomalies are coherent with an impaired B-T cells cooperation leading to disorganized Germinal Centres (GCs) and inadequate class-switched antigen-specific antibody responses as recently described in detail by Preite et al. and Bier at al. on mice models (12, 21, 22). These hyperplastic lymph nodes share a non-specific morphologic appearance with other reactive conditions. In some cases, morphology may resemble the EBV-associated polymorphous lymphoproliferative disorders typical of immunosuppressed post-transplant patients characterized by a polymorphic infiltrate of B and T cells, hyperplasia of monocytoid B-cell, epithelioid macrophages, light chain–restricted plasma cells and oligoclonal Ig gene rearrangement. Ig and TCR gene rearrangements tests usually reveal an oligo/polyclonality and EBER (*in situ* hybridizations of EBV-encoded RNA) evaluation often result positive in a small number of cells (2, 15). Despite Ig and TCR gene rearrangements analysis may rule out monoclonality (42) the correct histologic evaluation of these lesions is one of the main challenges in managing APDS patients. Thus, profound knowledge of this condition is requested to obtain a correct diagnosis and ensure a proper follow-up and the best therapeutic strategy.

Patients with APDS are usually treated with immunoglobulin replacement therapy and antibiotic prophylaxis to control infective complications. Immunosuppressive therapies aimed at control immune dysregulation are often needed. Among these, Rituximab could be a valid option since its ability to reduce non-neoplastic lymphoproliferation, control EBV proliferation and autoimmune disorders as well as autoimmune cytopenia (15). The hyperactivated PI3K-AKT-mTOR pathway, may also be targeted and modulated with various therapies. Rapamycin (mTOR inhibitor) or selective PI3Kδ inhibitors and Theophylline (acting as p110δ inhibitor), as reported in one patient (43), have proved to be well-tolerated and effective. Treated patients showed a reduction of incidence/severity of immune dysregulation manifestations and improved T and B phenotypes (28, 44–47). In particular, although Rapamycin does not entirely recover T and B cell defect, it has recently proved capable to restore NK function (13). Treated patients showed a reduction of lymphoproliferation but IBD and cytopenia responded less well. Moreover, one patient developed lymphoma during therapy (28). PI3K inhibitors have proved to improve lymphoproliferation, autoimmune and immune dysregulation symptoms as well as immunological alteration. It also proved to enhance the control of EBV chronic replication and viral lytic program *in vitro* (48). Careful long-term surveillance will allow to assess the efficacy and safety of such therapies. In a selected number of patients with refractory complications, hematopoietic stem cell transplantation (HSCT) could be a good option. Recently Dimitrova et al. reported 57 APDS1/2 patients undergoing HSCT with good results (2-year overall 86% and graft failure-free 68% survival probabilities). Graft failure was reported in particular if mTOR inhibitors were used in the first year post-HSCT probably due to an advantage to residual host cells (49–52).

**Figure 1:**
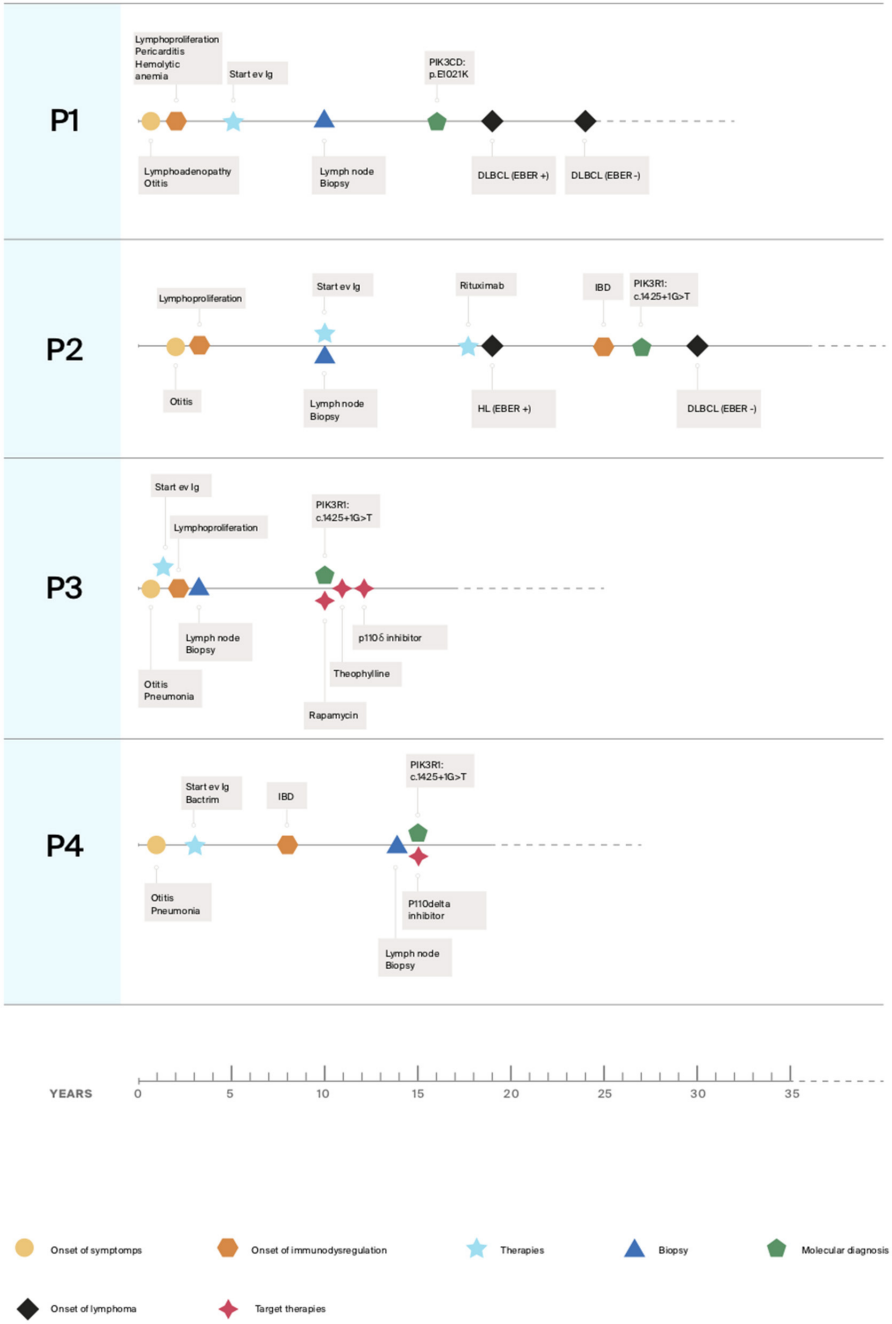
Clinical histories timelines.

Here we described our experience in the management of four patients, one with APDS1 (P1) and three with APDS2 (P2, P3, P4) presenting EBV chronic replication, immune dysregulation with lymphoproliferation and lymphoma. Clinical histories are resumed in Figure 1 and immunological phenotypes are reported in Table 1.

**Table 1:** Immunological characterization of patients.

	**P1 (6 y)**	**P1 (24 y)**	**P2 (17y)**	**P2 (34 y)**	**P3 pre T** **(11 y)**	**P3 post T (14 y)**	**P4 pre T** **(15 y)**	**P4 post T (17 y)**
Total Lymphocyte count 10^3^/ul	1,693 (1,200–4,700)	**1,280** (1,400–4,200)	1,440 (1,400–4,200)	1,950 (1,200–4,100)	1,950 (1,400–4,200)	1,710 (1,400–4,200)	**1**,**280** (1,400–4,200)	**1**,**150** (1,200–4,100)
CD3+ CD45+	72 (55–97)	79.6 (50–91)	90.6 (50–91)	78.5 (50–91)	**96.2** (52–90)	**94.7** (52–90)	**92.5** (52–90)	90 (50–91)
CD3– CD16+CD56+	11 (2–31)	9 (5–49)	5 (5–49)	12.6 (5–49)	**1.84** (4–51)	**3.1** (4–51)	4.4 (4–51)	6.9 (5–49)
CD3+ CD4+	**20** (26–61)	**21.5** (28–64)	**24.5** (28–64)	**11.7** (28–64)	**19.4** (20–65)	20.1 (20–65)	21.2 (20–65)	29 (28–64)
CD3+ CD8+	46 (13–47)	**57.2** (12–40)	**51.8** (12–40)	**58** (12–40)	37.6 (14–40)	**58.8** (14–40)	**56.4** (14–40)	**48.6** (12–40)
CD19+ CD45+	13 (9.8–17.72)	9.2 (6.6–10.8)	**1.3** (10.2–15.4)	**5.1** (7.2–11.2)	**2.4** (10.2–15.4)	**1.3** (10.2–15.4)	**2** (10.2–15.4)	**2.1** (10.2–15.4)
**% CD3 lymphocyte subsets**								
TCRα/β+		98.8 (44–92)		90 (36–98)	81.7 (39–92)	79 (39–92)	87.1 (39–92)	92.5 (36–98)
TCRγ/δ+		**1.15** (2–24)		9.4 (0.83–11)	16.1 (2–17)	**21** (2–17)	11.7 (2–17)	7.42 (0.83–11)
CD4– CD8–		0.5 (0.57–5)		2.7 (0.57–5)	**0.49** (0.54–6)	0.9 (0.54–6)	**6.82** (0.54–6)	**6.42** (0.57–5)
**% CD4 lymphocyte subsets**								
CD27+CD45RA+ Naïve		**4** (16–100)		**9.2** (16–100)	**18.8** (25–63)	**25.8** (31–57)	**16.6** (31–57)	**20.3** (31–57)
CD31+CD45RA+ (RecentThymicEmigrants, RTE)		**2** (7–100)		11.5 (7–100)	**16.7** (43–67)	**24.7** (37–62)	**11.7** (37–62)	**13** (37–62)
CD27+CD45RA– (CentralMemory)	76	72.1 (18–95)	91.8	76.7 (18–95)	**71.3** (11–25)	**53.4** (10–27)	**73.1** (10–27)	**70.3** (10–27)
CD27–CD45RA– (EffectorMemory)		23.5 (1–23)		9.88 (1–23)	9.6 (12–30)	15.2 (12–44)	10.1 (12–44)	9.25 (12–44)
CD27–CD45RA+ (EffectorMemoryCD45RA+, EMRA)		0.35 (0.0031–1.8)		**4.23** (0.0031–1.8)	0.26 (4–24)	5.6 (4–12)	0.2 (4–12)	0.0791 (4–12)
CD25+CD127lowFOXP3+ (RegulatoryTcell, Treg)		3.93 (4–17)		0.32 (4–17)		5.49 (4–20)		9.55 (4–17)
CD45RO+ CXCR5+ (Follicular helper T cell. Tfh)		7.4 (5–56)		13.2 (5–56)		10.1 (7–47)		8.6 (5–56)
**% CD8 lymphocyte subsets**								
CCR7+CD45RA+ (Naïve)		**2.6** (6–100)		**0.6** (6–100)	**2.4** (22–58)	**2.72** (18–61)	**3.1** (18–61)	**5.7** (18–61)
CCR7+CD45RA– (Central Memory)	41	**0.5** (1–20)	82.6	**0.8** (1–20)	**0.79** (2–15)	**0.196** (3–12)	**0.5** (3–12)	**0.394** (3–12)
CCR7–CD45RA– (EffectorMemory)		68.8 (14–98)		83 (14–98)	**62.7** (24–58)	53 (25–58)	**81.6** (25–58)	**88.8** (25–58)
CCR7–CD45RA+ (EffectorMemoryCD45RA+. EMRA)		28.1 (7–53)		15.6 (7–53)	**44.1** (7–26)	**44** (5–20)	14.8 (5–20)	5.1 (5–20)
**% B lymphocyte subsets**								
CD24++CD38++ (Transitional)	**59** (4.5–9.2)	**52** (3–5.9)	**60** (3.9–7.8)	**18.3** (1–3.6)	**51.5** (3.9–7.8)	**59.5** (3.9–7.8)	**60.8** (3.9–7.8)	2.8 (3.9–7.8)
CD27–IgD+IgM+ (Naïve)	**64** (69.4–80.4)	76.6 (65.6–79.6)	**68.6** (75.2–86.7)	73.4 (58–72.1)	**57.5** (75.2–86.7)	84.1 (75.2–86.7)	**19.4** (75.2–86.7)	78.9 (75.2–86.7)
CD27+IgD+IgM+ (Unswitched memory)	11 (7.5–12.4)	10.6 (7.4–13.9)	**2.4** (4.6–10.2)	**6.4** (13.4–21.4)	**1.34** (4.6–10.2)	**1.7** (4.6–10.2)	5.2 (4.6–10.2)	**3.09** (4.6–10.2)
CD27+IgD–IgM– (Switched memory)	7.4 (5.2–12.1)	**5.1** (7.2–12.7)	7.2 (3.3–9.6)	**9.05** (9.2–18.9)	**2.6** (3.3–9.6)	**2.7** (3.3–9.6)	6.3 (3.3–9.6)	5.29 (3.3–9, 6)
CD27– IgD– IgM–		**7.7** (2.1–4.4)		**11.1** (2.1–5.3)	**38.5** (2.3–5.5)	**11.3** (2.3–5.5)	**69.1** (2.3–5.5)	**12.8** (2.3–5.5)
CD21low CD38low		1.3 (0.9–3.1)		4.04 (1.8–4.7)	**5.58** (0.9–3.3)	**4.06** (0.9–3.3)	2 (0.9–3.3)	0.91 (0.9–3.3)
**% NK cell subsets**								
CD56 bright				14.6 (4.1–12.9)		32.8 (4.1–12.9)		21.2 (4.1–12.9)
CD56 dim				78.5 (79.8–91.0)		55.6 (79.8–91.0)		77.1 (79.8–91.0)
CD56 neg				7.2 (3.6–8.0)		11.1 (3.6–8.0)		2.18 (3.6–8.0)
IgG (mg/dl)		727^*^	725^*^	608^*^	883^*^	827^*^	757^*^	721^*^
IgA (mg/dl)		48	**<5**	**<5**	**<5**	**<5**	**<5**	**<5**
IgM (mg/dl)		**326**	**749**	**202**	**209**	**377**	**326**	**204**
EBV (copies/ml)		66.276	<100	343	3.1869	3.635	1.146	571
CMV (copies/ml)		neg	neg	neg	neg	neg	neg	neg
HHV6 (copies/ml)		neg	neg	neg	<500	626	<500	<500

* *During Ig replace therapy. Range value in the bracket (53–55)*.

*With bold values we highlight values out of the normal range*.

Written informed consent was provided by patients for the publication of these case reports.

## Cases Description

**Figure 2:**
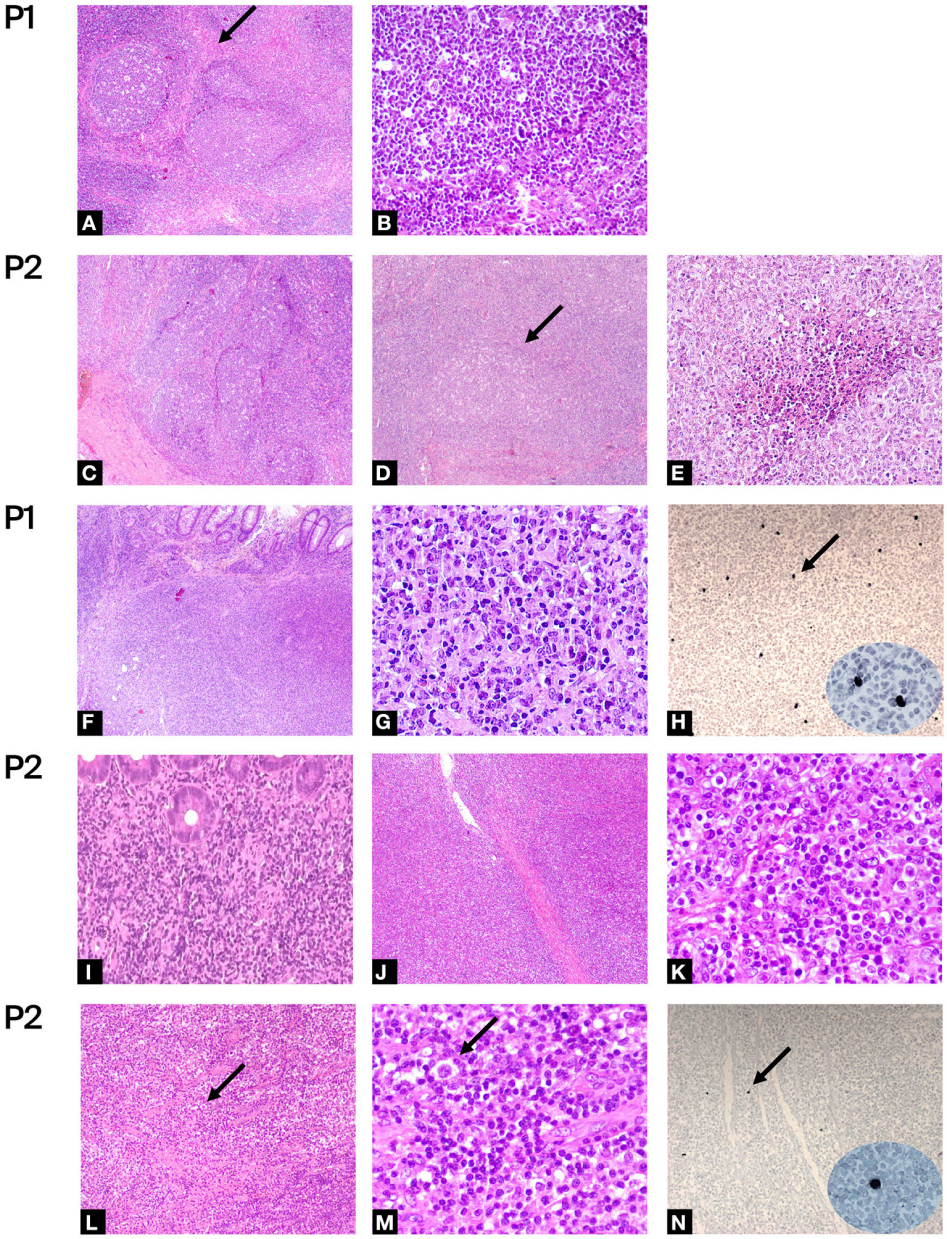
Histologic evaluations: **(A–E)** Hematoxylin-eosin of benign lymph node biopsy of P1 and P2. Figures at low magnification shows: **(A,C,D)** large and irregularly expanded “naked” GC with ill-defined outlines, loss of the mantel zone and polarization, monocytoid B-cell hyperplasia, numerous large histocyte and tingible bodies (**A,C**:4×, **D**: 5×); **(A)** the arrow shows area of sclerosis around the follicle. Figures at higher magnification shows: **(B)** absence of mantel zone surrounding the germinal center (40×); **(E)** foci of necrosis (20×). **(F–N)** Hematoxylin-eosin of malignant biopsy of P1 and P2. **(F,G,I)** infiltration by polymorphous DLBCL of large bowel (**F**:4×, **I**:20×, **G**: 40×) and **(J,K)** of lympho-node (**J**: 4×, **K**:40×). **(L,M)** low (10×) and high (40×) magnification of lympho-node of P2 infiltered by NLPHL. Arrows show “popcorn” cells with multilobate nucleus and scant cytoplasm surrounded by rosetting activate small lymphocytes. **(H, N)** *In-situ* hybridization for EBER, arrow shows some positive cells (×10, in ellipse high magnification ×40).

P1 is a 24 years old Romanian male, followed since 5 years old for CID and definitively diagnosed at 16 years old as APDS1 by whole-exome sequencing (WES) (PIK3CD: p.E1021K). He received with good tolerance all routine vaccinations in Romania (BCG included). In the first years of life, he presented a lymphadenopathy diagnosed and treated as toxoplasmosis. Since early life, he presented recurrent sinopulmonary infections, otitis with subsequent need for trans-tympanic drainage, recurrent lymphadenopathy, an episode of hemolytic anemia and three episodes of pericarditis. Immunological investigations showed progressive lymphopenia and impaired B and T subsets leading to a diagnosis of CID (Table 1). As previously reported a normal frequency of bona-fide T-regs and distribution of other T-reg subsets (resting, activated, and non-suppressive) and a defect in the maturation and function of myeloid cells with a reduced capability of monocyte-derived macrophages (MDM) to control the intracellular mycobacterial activity *in vitro* were found (14). A chronic EBV infection with incomplete seroconversion (absent EBNA-IgG) was documented. Since the diagnosis of CID he has been treated over time with immunoglobulin (Ig) replacement therapy, respiratory therapy, cycles of antibiotics, steroids and Rituximab with discrete control of infections, lymphoproliferation and autoimmune manifestations. During follow-up he presented several episodes of lymphadenopathy requiring whole biopsy. The main histologic finding was an atypical follicular hyperplasia characterized by voluminous “naked” GC with ill-defined contours, marked reduction of mantle zone and prominent monocytoid B cells reaction (Figures 2A,B). At 19 years old, he presented fatigue weight loss and diarrhea together with multiple lymphadenopathies. Lymph node and endoscopic duodenal mucosa biopsies revealed a diffuse large B cell lymphoma (DLBCL) (Figures 2F,G). Sporadic cells resulted EBER-positive (Figure 2H). He received 3 R-CHOP (Rituximab, Cyclophosphamide, Doxorubicin, Vincristine, Prednisolone) + 2 CHOP chemotherapy cycles. After 4 years he developed a further episode of pericarditis and received a concurrent diagnosis of atypical mycobacterial pulmonary infection on a bronchoalveolar lavage following a thorax-CT showing multiple bilateral nodular and inflammatory opacities. A routine follow-up endoscopy revealed a caecal ulcerated polypoid lesion. The histology was diagnostic for a DLBCL, EBER evaluation resulted negative. He was treated with a combination of reduced toxicity chemotherapy with Rituximab, Ibrutinib and Bendamustine.

P2 is a 36 years old female diagnosed for APDS2 at 27 years old by Sanger sequencing (PIK3R1: c.1425+1G>T). She was previously described in 2006 as hyper-IgM with growth and pubertal disturbances (56). She was admitted for failure to thrive, recurrent otitis and severe infections requiring hospitalization since early childhood. The severe lymphoproliferation, in particular of the oral lymphoid tissue, led to two tonsillectomies during the first decade. Due to recurrent lymphadenopathy, she underwent repeated biopsies. At the age of 8 years, the histologic evaluation of a cervical lymph node showed a distortion of architecture due to large, irregularly expanded “naked” GC. Foci of necrosis were also present (Figures 2C–E). A diagnosis of infectious lymphadenitis was made but no infectious agents were identified. At the age of 10, she was diagnosed with hypogammaglobulinemia and hyper-IgM and started immunoglobulin replacement therapy. A persistent EBV replication was documented. Immunologic phenotype is reported in Table 1. During follow-up, The CT scan performed at 17 years of age revealed a thickening of the distal colon with several mesenteric enlarged lymph nodes. The histologic evaluation showed a polyclonal B lymphoproliferation EBV negative. Due to the persistence of lymphadenopathies associated with chronic EBV replication she was treated with three Rituximab infusions with a complete virologic and clinical remission. One year later, at 18 years old, after the recurrence of cervical lymphadenopathy, she received a diagnosis of a nodular lymphocyte-predominant Hodgkin Lymphoma (NLPHL) (Figures 2L,M). A minority of cells resulted EBER-positive (Figure 2N). She was treated with 3 ABVD (Adriamycin, Bleomycin, Vinblastine, Dacarbazine) chemotherapy cycles and radiotherapy. At 25 years old she developed abdominal pain associated with persistent diarrhea and bloody stools. The repeated endoscopies confirmed an IBD. After the molecular diagnosis, at 30 years old, endoscopic follow-up performed during the enrollment evaluations for the PI3K inhibitors trial showed a bulging of duodenal mucosa and widespread pseudopolypoid lesions of the colon. The histologic examination of bowel biopsy and lymph nodes revealed a DLBCL developed in the context of lymphatic hyperplasia (Figures 2I–K). EBER evaluation resulted negative. She was treated with five cycles of R-CHOP plus two Rituximab infusions. The subsequent endoscopic evaluation showed the persistence of IBD treated with budesonide with good control of intestinal symptoms.

P3 is a 17 years old female diagnosed for APDS2 at 10 years old by WES (PI3KR1: c.1425+1G>A exon skipping mutation). She was born late preterm at 34 weeks and 2 days in good clinical conditions for abnormal uterine artery doppler flow. She was hospitalized at 8 months old for bronchiolitis complicated by pneumonia. At the age of one, she was diagnosed with combined immunodeficiency (CID) with hyper IgM, and started immunoglobulin replacement therapy. Since the age of two, she showed recurrent episodes of lateral neck lymphadenopathy and infections. In particular, she suffered of pneumonia and otitis with subsequent hypoacusia and need for trans-tympanic drainage and a growth. At 3 years old she underwent adenotonsillectomy and a whole lymph node biopsy which excluded a malignancy. A chronic EBV infection was detected. At 9 years old a thorax CT scan showed a relevant enlargement of mediastinal lymph nodes causing a middle lobe syndrome. At 10 years old, she started suffering from dysphonia and dysphagia caused by severe hyperplasia of the lingual tonsils treated with steroids and antibiotics. She started Rapamycin with partial control of proliferation but without improvement of vocal function. Due to recurrent headaches, Rapamycin was progressively replaced with Theophylline with a significant reduction of lymphadenopathies resulting in voice restoration and a gradual improvement of her quality of life and scholastic performance with only sporadic headache episodes over 1 year. Immunologic laboratory investigation showed a reduction of IgM and a mild improvement of phenotype (a slight increase of CD4+CD45RA+CD31+ and CD27+B cells) associated with a reduction of infections requiring antibiotic therapy (43). Afterward, she started a specific target therapy using the PI3K inhibitors (see below).

P4 is a 19 years old male diagnosed for APDS2 at the age of 15 by Sanger Sequencing (PIK3R1: c.1425+1 G >A). Since the age of 1 year he presented recurrent respiratory infections treated with antibiotic therapy 2 or 3 times a month. At 3 years old, he was diagnosed with CID and hyper-IgM. He started immunoglobulin replacement therapy and cotrimoxazole prophylaxis with benefits. At the age of eight by the appearance of persistent diarrhea, weight loss and reduction of IgG levels he was treated with azithromycin. Despite the microbiologic stool evaluation were negative he showed a temporary improvement of symptoms. The endoscopic bowel examination performed revealed lymphoid hyperplasia. He was treated with steroid therapy tapered in 1 year with a resolution of symptoms. At the age of 14 years, he developed evident splenomegaly and lymphadenopathy of lateral-cervical, axillary and inguinal lymph nodes. At the histologic evaluation, a mixed B and T lymphocytic inflammatory infiltrate and a chronic EBV infection were detected. Following the persistence of important lymphoproliferation a new lateral cervical whole lymph node biopsy was done. After that a tracheostomy was performed due to the severity of airway obstruction by hyperplasia of the lingual tonsils and lymphadenopaty. The histologic evaluation showed a B cell hyperplasia, the absence of GCs and of the mantle zone, sparse IgM+ plasma cells. EBER *in situ* hybridization was negative. An upper/bowel endoscopic evaluation excluded a malignant lymphoproliferative involvement. Thanks to the APDS2 molecular diagnosis he started a specific target therapy with PI3K inhibitors.

**Figure 3:**
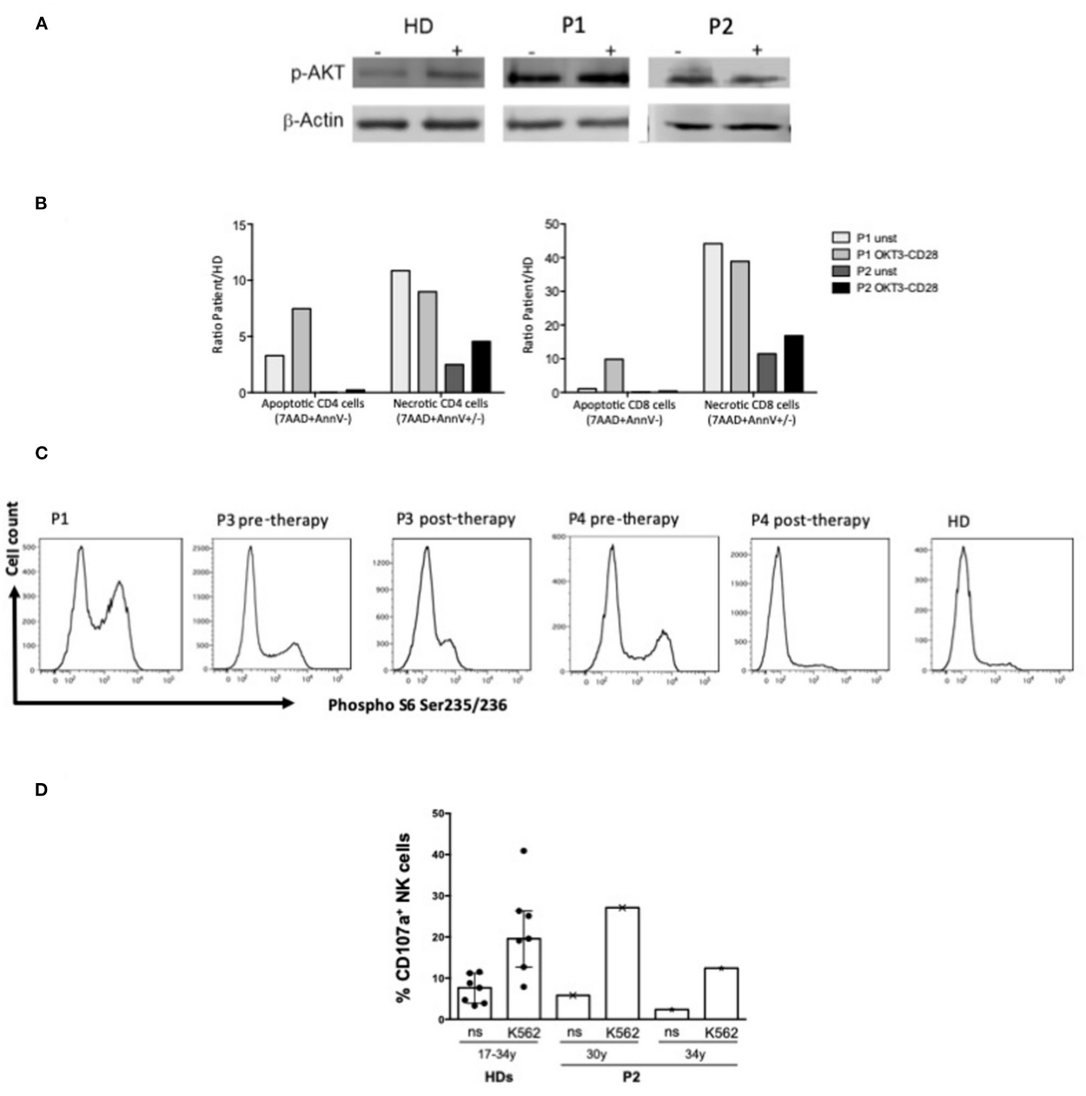
Functional analysis. **(A)** Western blot analysis of p-Akt and β-actin protein expression in T blast from P1 (APDS1), P2 (APDS2) and HD with or w/o OKT3-CD28 stimulation (10 min); **(B)** Percentage of apoptotic (7AAD-AnnexinV+) or necrotic (7AAD+AnnexinV ±) CD4 and CD8 gated cells from APDS1 (P1) and APDS2 (P2) patients with or w/o 48 h of OKT3-CD28 stimulation. Results were showed as fold increase calculated as ratio between patients and HD. **(C)** *Ex vivo* phospho-S6 Ser235-236 intracellular staining performed on whole blood in CD3+ gated lymphocytes from P1 (APDS1) and P3 and P4 (APDS2) pre and post therapy with PI3K inhibitor and a healthy donor (HD). **(D)** Bar plots represent pattern of CD107a expression measured by flow cytometry on gated NK cells of an APDS2 patient at two time points (30 and 34 years of age) and of HDs (*n* = 7, age ranging from 17 to 34 years) following 6 h culture of PBMCs (effectors, E) with and without (not stimulated, ns) K562 cell targets (T) at an E:T ratio of 10:1. The median ± IQR is reported for HDs.

P3 and P4 were enrolled in a phase 1b open-label study and treated with the PI3K inhibitor Seletalisib (UCB585). They showed a well-tolerance, improved immune phenotype (Table 1), functional test (Figure 3C), clinical symptoms and quality of life (46). The therapy was discontinued after 2 years by the decision of the Company. In the last 2 years, P3 and P4 received compassionate Leniolisib. Unfortunately, P3 could not tolerate more than 30 mg of Leniolisib daily due to the occurrence of painful ulcers in the mouth and lips. The addition of 1 mg daily of Colchicine resulted in improved control of aphthous symptoms and overall clinical conditions, allowing administer 30 mg of Leniolisib twice daily. She also continued prophylaxis with cotrimoxazole 3 days per week, while she could reduce her need for immunoglobulins to 4 g every 10 days (230 mg/kg/4 weeks).

As reported in Table 1 patients' immune characterization showed a reduction of CD4+ T cells, particularly CD4+ Naïve and an expansion of terminally differentiated CCR7–CD45RA+ effector memory CD8+ T cells. B cells phenotype was characterized by a marked expansion of transitional B cells and a reduction of memory and switched memory B cells and by a reduction of total CD19+ B cells, in particular in APDS2 patients (P2, P3, P4). As previously reported NK cells of P1 showed a defective cytotoxic activity against K562 cell line and a high expression of senescence-associated marker CD57 (14). NK cells had a normal frequency and distribution in P2 and P4, whereas P3 displayed low NK cell frequency and expanded CD56bright cells at the expense of CD56dim cells compared with HDs (Table 1). IN P2 the NK cell cytotoxicity resulted similar to that of HD controls at two different time points (Figure 3D).

Western blot of pAKT of P1 and P2 (Figure 3A) and flow cytometry analysis of Phospho S6 Ser235/236 of P1, P3, P4 (Figure 3C) showed the hyperactivation of the PI3K-AKT-mTOR signaling pathway. In P3 and P4 cytometry analysis showed a reduction of Phospho S6 Ser235/236 after the treatment with Seletalisib (Figure 3C). *In vitro* CD4+ and CD8+ T cells resulted more apoptotic and necrotic after stimulation with anti-CD3/CD28 respect to the unstimulated condition and the HD control (P1, P2, Figure 3B).

## Discussion

This cases series shows the difficulties in the management of APDS1/2 patients. All patients were initially diagnosed with a CID, hypogammaglobulinemia and hyper IgM (Table 1). In P1 and P3 the genetic diagnosis was obtained by WES while in P2 and P4 by Sanger Sequencing and confirmed with functional tests (pAKT WB and pS6, Figures 3A,C) which showed the hyperactivation of the PI3K-AKT-mTOR signaling pathway.

According with literature patients' immune characterization showed a skewed distribution of T and B cells with a marked expansion of transitional B cells at the expense of the memory and switched memory B cells, and a reduction of T CD4+ Naïve and an expansion of terminally differentiated CCR7–CD45RA+ effector memory CD8+ T cells (Table 1). As we previously described T cells *in vitro* resulted more apoptotic and necrotic after stimulation with anti-CD3/CD28 respect to the unstimulated condition and the HD control (P1, P2 Figure 3B). Moreover, NK cell cytotoxicity was reduced in P1 (14), in line with other studies showing functional impairment of NK cells in APDS1 (13) P3 displayed low NK cell frequency and expanded CD56bright cells while in P2 NK cells had normal frequency and distribution (Table 1) and the NK cell cytotoxicity resulted similar to that of HD controls (Figure 3). To our knowledge, this is the first reported evidence that the cytotoxic function of NK cells is maintained in APDS2 patients. It should be noted that the first of these analyses was performed few months after chemotherapy (R-CHOP) that could influence NK activity and the second one after few years (57). Therefore, it will be interesting to extend analyses of NK cell cytotoxicity to other APDS2 patients not treated with chemo/immunosuppressive therapies.

All patients developed recurrent respiratory tract and a chronic EBV infection (Table 1) in line with data from the literature (15, 16). P1 was diagnosed with an atypical mycobacterial pulmonary infection when he was 23 during an episode of pericarditis. He had been vaccinated at birth with BCG with referred good tolerance, although he developed a controversial adenomegaly in the first year of life diagnosed from another Center as toxoplasmosis. A susceptibility for toxoplasma infection is reported in patient with APDS (58). The occurrence of a subsequent atypical mycobacterial infection and the susceptibility already reported by Coulter et al. (15) such as persistent granulomatous skin lesions after BCG vaccination, underlie the importance of screening these pathogens. As assessed *in vitro* selective p110δ inhibitors may restore the ability to control mycobacterial infections by monocyte-derived macrophages of APDS patients (14). It will be interesting to evaluate the incidence and evolution of mycobacterial infection in treated patients.

All patients developed recurrent episodes of lymphadenopathy and two patients (P2 and P4) symptomatic lymphoproliferative colitis requesting repeated whole lymph nodes and endoscopic biopsies. Two patients (P1, P2) were diagnosed with lymphoma involving the large bowel. P1 presented diarrhea and weight loss at the first DLBCL diagnosis and was asymptomatic at the relapse. P2 was diagnosed for DLBCL during the endoscopic follow-up for the IBD, in the absence of symptoms, as enrollment evaluations for the PI3K inhibitors trial. This highlights as a strict follow-up including endoscopic evaluations is fundamental for APDS1/2 patients even in the absence of specific symptoms. The analysis shows some EBER positive cells in both biopsies diagnostic for lymphoma at the first diagnosis (P1 and P2, Figures 2H,N) that can only be considered as a marker of the previous infection. At the time of relapse, EBER resulted negative on the neoplastic population in both patients. Thus, as previously described, the EBV infection is an important trigger but is not the only player in lymphoma pathogenesis (15, 16, 27, 38).

After the APDS2 diagnosis, P3 and P4 were enrolled in a phase 1b open-label study and treated with PI3K inhibitors (Seletalisib, Leniolisib) with good tolerance and improvement of symptoms and quality of life (46). These therapies represent a good opportunity, improving the outcome, the immunologic phenotype and functional tests (P3 and P4: Table 1 and Figure 3C), reducing the risk for immune dysregulation manifestations, the severity of lymphoid proliferation and hopefully the risk for a switch to monoclonal proliferation (28, 43–46). Unfortunately, owing to the study exclusion criteria, P1 and P2 could not be enrolled in the study for treatment with PI3K inhibitors due to the recent history of lymphoma. Besides, increased genomic instability in B cells treated with selective PI3Kδ inhibitors has been recently described *in vitro* explained by an augmented frequency of AID-induced translocation to IGH and AID off-target sites (59). Both of them relapsed during follow-up. At the lymphoma relapse, P2 was treated with personalized chemotherapy in order to reduce toxicity after an unsuccessful search for a suitable HSCT donor. Obtain a genetic diagnosis and subsequently the proper histologic evaluation of lymphadenopathies are essential steps to offer a personalized treatment and improve the outcome for these patients. Considering the indolence and persistence of the lymphoproliferation, which frequently mimic a malignancy and only sometimes become aggressive, early personalized target therapies involving specific inhibitors and Rituximab should be considered. In this perspective it is crucial to identify prognostic markers that lead therapeutic choices. With this purpose the International ESID-APDS European Registry will allow assessing the long-term follow-up and the natural history of this rare and heterogenic disease particularly after the introduction of PI3K inhibitors.

## Conclusions and Future Directions

One of the main challenges in the management of APDS1/2 patients is to set the proper follow-up and make a correct histologic evaluation of persistent and recurrent lymphoproliferation and lymphadenopathy. Periodic laboratory and upper/low endoscopic evaluations must be scheduled, even without specific signs and symptoms. Repeated microbiologic screening, including the search for EBV and mycobacterial infections, whole lymph nodes/endoscopic biopsies and a multidisciplinary team discussion are fundamental to achieve the proper diagnosis and choose specific personalized treatments. These treatments may be helpful in controlling the immune dysregulation manifestations, recurrent/chronic infections (i.e., EBV) and in the reduction of chemotherapy toxicity, also in view of potential treatment for relapsed malignancies or HSCT. New clinical trials and extended studies will allow testing the efficacy and security of these specific therapies, the best timing and the opportunity to treat also APDS1/2 patients with a history or diagnosis of lymphoma. Further studies are needed to elucidate the molecular mechanisms responsible for the impaired homeostasis, activity and interaction of the different leukocyte subsets and their role in lymphoproliferation/immune dysregulation manifestations. Moreover, international multicentric studies are needed to define the natural history of this rare disease. Both will allow to identify new markers of the disease, applicable to guide treatment choices, and targets for personalized immunomodulant therapies.

## Data Availability Statement

The raw data supporting the conclusions of this article will be made available by the authors, without undue reservation.

## Ethics Statement

Written informed consent was provided by patients for the publication of these case report.

## Author Contributions

BR and DA participated in writing the paper. GD, MC, SD, CCi, MDe, and MDo participated in the investigation, performing the genetic analysis, immunological experiments, and the formal analysis of the article. RD performed the histologic analyses and participated in formal analysis of the article. CM, CG, FC, LP, and VS participated in data curation and were involved in the clinical care of the patients. AF, PP, and IQ participated in the validation of the article. CCa, ATo, and ATr participated in project administration, supervised the work, and reviewed and edited the article. All authors have read and agreed to the published version of the manuscript.

## Conflict of Interest

The authors declare that the research was conducted in the absence of any commercial or financial relationships that could be construed as a potential conflict of interest.

## Publisher's Note

All claims expressed in this article are solely those of the authors and do not necessarily represent those of their affiliated organizations, or those of the publisher, the editors and the reviewers. Any product that may be evaluated in this article, or claim that may be made by its manufacturer, is not guaranteed or endorsed by the publisher.
